# PET/CT and exome sequencing in late onset multiple acyl-CoA dehydrogenase deficiency: a case series and literature review

**DOI:** 10.1186/s12920-025-02210-8

**Published:** 2025-10-21

**Authors:** Dong-Fang Lin, Huan Sheng, Qiang Qu, Ze-Tao Liao

**Affiliations:** 1 Department of Rheumatology, the Third Affiliated Hospital of Sun Yat-sen University, Guangzhou, Guangdong Province China; 2 Department of Rheumatology, Lujiang County Hospital of Traditional Chinese Medicine, Hefei, Anhui Province China; 3 Deputy Dean, School of Journalism and Communication, Minzu University of China, Beijing, China

**Keywords:** Multiple acyl-CoA dehydrogenase deficiency, Myopathy, Positron emission tomography/Computed tomography, Exome sequencing, Riboflavin

## Abstract

**Background:**

Multiple acyl-CoA dehydrogenase deficiency (MADD) is a rare autosomal recessive disorder. Riboflavin-responsive MADD (RR-MADD) represents a treatable subtype, though its molecular mechanisms are incompletely characterized.

**Case presentation:**

Two patients presented to department of Rheumatology, the 3rd Affiliated Hospital of Sun Yet-sen University with progressive proximal muscle weakness. A 2-fluorine-18-fluoro-2-deoxy-D-glucose positron emission tomography/computed tomography (^18^F-FDG PET/CT) scan revealed marked metabolic hyperactivity in cervical and paraspinal muscles. Histopathology confirmed lipid storage myopathy, and exome sequencing (ES) identified electron transfer flavoprotein dehydrogenase gene (*ETFDH*) mutations. Both patients harbored the common variant NM_004453.4:c.250G > A (p.Ala84Thr). Case 1 exhibited homozygosity for this variant and potential XYY syndrome, while Case 2 carried a compound heterozygous mutation including a novel frameshift variant, NM_004453.4: c.265_266del (p. Gln89Valfs*6), and concurrent SLC25A13 mutation linked to adult-onset citrullinemia type II. Riboflavin supplementation achieved complete biochemical remission in both cases. Retrospective analysis for previous reports of homozygous single-locus missense *ETFDH* gene mutations revealed that patients with mutations in the flavin adenine dinucleotide binding domain (FAD) or the iron-sulfur cluster domain (Fe-S) exhibited a better response to riboflavin and better prognosis.

**Discussion:**

^18^F-FDG PET/CT is a promising tool for evaluating myopathic involvement in MADD. ES enables rapid and comprehensive diagnosis of MADD and detection of coexisting genetic disorders. The prognosis is related to mutation forms, mutation pathogenicity, and the located structural domain.

**Supplementary Information:**

The online version contains supplementary material available at 10.1186/s12920-025-02210-8.

## Introduction

Multiple acyl-CoA dehydrogenase deficiency (MADD), also termed glutaric aciduria type II, results from defects in electron transfer flavoprotein (ETF) or ETF-ubiquinone oxidoreductase (ETF-QO). UniProtKB identifiers: Q16134) [1]. This disrupts mitochondrial β-oxidation, leading to toxic metabolite accumulation [2]. Clinically, MADD is classified into neonatal-onset forms (types I/II) and late-onset type III [3]. Types I and II are typically fatal in the neonatal period, while type III patients may present with progressive proximal myopathy, fatty liver, neuropathy, intermittent episodes of vomiting, hypoglycemia. or metabolic acidosis [4]. Type III includes riboflavin-responsive MADD (RR-MADD) [5, 6] and non-RR-MADD. More than 80% of type III cases are RR-MADD [7].

ETF-QO is a 64 kDa monomeric protein present in the mitochondrial inner membrane, and contains three major domains: (1) flavin adenine dinucleotide-binding domain (FAD); (2) iron-sulfur cluster domain (Fe-S); and (3) ubiquinone Q-binding domain (UQ). ETF-QO has two redox centers: Fe-S and FAD. ETF-QO oxidizes ETF, and the electrons from ETF enter ETF-QO through the FE-S. Flavin adenine dinucleotide then transports the electrons through the protein, and the electrons reduce ubiquinone Q. Lastly, ubiquinone Q transfers the electrons to the bc1 complex, and the mitochondrial electron transfer chain finally produces adenosine triphosphate (ATP) [1, 2]. Disorders in the above process led to obstruction of the electron transport chain and β-oxidation pathway in mitochondria, subsequently resulting in excessive lipid accumulation. The inability to oxidize fatty acids prevents the synthesis of ketone bodies, an essential alternate energy source for the heart [3]. The build-up of toxic metabolites and heightened oxidative stress cause secondary damage to the mitochondrial oxidative phosphorylation complexes, reducing ATP synthesis and ultimately impairing the function of organs [4, 5].

ETF is known to be a heterodimer of alpha (30 kDa) and beta (28 kDa) subunits. The genetic defects in *ETFɑ* and *ETFβ* encoding ETF, and defects in the electron transfer flavoprotein dehydrogenase gene (*ETFDH*) encoding ETF-QO are responsible for MADD. Reports to date suggest that *ETFDH* defects account for 70–93% of all MADD cases [6, 7]. However, the severity of symptoms is currently not considered to be directly related to the above genes (*ETFɑ*,* ETFβ*, or *ETFDH*), but more likely related to the nature and location of the mutation [8]. *ETFDH* is located at 4q32.11 and has 13 exons. So far, more than 300 mutations in *ETFDH* have been reported according to the Human Gene Mutation Database (HGMD), including point mutations, nonsense mutations, insertions, deletions, and splicing mutations. Three hotspot variants predominate in China, but area rarely seen outside of China: NM_004453.4:c.250G > A (p.Ala84Thr) in patients from southern China, NM_004453.4:c.770 A > G (p.Try257Cys) and NM_004453.4:c.1227 A > C (p.Leu409Phe) in patients from northern China [9–12].

The initial symptom of the two cases reported herein was muscle weakness. It was found that 2-fluorine-18-fluoro-2-deoxy-D-glucose positron emission tomography/computed tomography (^18^F-FDG PET/CT) can help differentiate fatty acid deposition myopathy in MADD from other myopathies. The two *ETFDH*-deficient cases presented herein were from southern China and had a NM_004453.4: c.250G > A (p. Ala84Thr) mutation as mentioned, but one of them had a frameshift mutation, NM_004453.4: c.265_266del (p. Gln89Valfs*6). In addition, the use of the next-generation exon sequencing technology instead of traditional *ETFDH* single nucleotide primers for polymerase chain reaction (PCR) sequencing indicated that both cases might have other hereditary diseases. This provides a new insight into the unclear association between genotype and phenotype of late-onset MADD. Finally, protein domain analysis of previous case reports of late onset MADD patients with homozygous single-locus missense mutations in *ETFDH* showed that the better prognosis for RR-MADD was associated with mutations located in the FAD and Fe-S regions of *ETFDH*.

## Case presentation

### Case 1

A 29-year-old male presented with a 4-year history of progressive lower limb and neck weakness, post activity wheezing, and intermittent abdominal pain. He denied vomiting, diarrhea, fever, and cough. Prior evaluations revealed a persistently elevated creatine kinase level (CK: 1,800–3,000 U/L). Electromyography testing supported muscle involvement. A muscle biopsy was performed, and microscopic examination after hematoxylin and eosin staining suggested myositis. With the possibility of polymyositis (PM) in consideration, methylprednisolone, globulin, methotrexate, hydroxychloroquine, and other treatments were prescribed at a local hospital before transfer to ours due to the poor effects of the treatments, and his CK level had increased to 2,317 U/L 2 weeks before admission to our hospital.

With respect to family history, his mother died of breast cancer and never experienced any muscle pain or muscle weakness before her death. Additionally, no other family members including the patient’s father, younger sister, and elder sister, and the elder sister’s 4-year-old daughter never had any problems with muscle weakness or paint. The CK levels of all the family members were normal at routine examinations.

On admission, physical examination showed the patient had a normal level of consciousness, vital signs, intelligence, tenderness in the cervical muscles and proximal muscles of upper and lower limbs, and no hepatosplenomegaly. His neurological examination was normal, but his muscle strength of neck flexors, neck extensors, and proximal muscles of upper and lower limbs were decreased based on manual muscle testing (MMT) (rated as 3, 3, 4+, and 4-, respectively), as shown in Table 1. Routine blood tests revealed elevated liver and muscle enzymes (Table 2). His aspartate transaminase (AST) was 273 IU/L (normal range 15–40 IU/L); alanine transaminase (ALT) was 187 IU/L (normal range 3–35 IU/L); lactate dehydrogenase (LDH) was 2364 IU/L (normal range 3–35 IU/L); and CK was 2,282 IU/L (normal 24–184 IU/L). Antinuclear antibodies (ANA), the extractable nuclear antigen (ENA) series, and myositis antibody spectra were all negative.

**Table 1:** Manual muscle testing (MMT) scale of the two patients on examination

No.	neck		proximal upper limbs			proximal lower limbs			distal limbs
	flexion	extensor	deltoid	biceps	triceps	iliopsoas	gluteus medius	gluteus maximus	
1	3	3	4+	4+	4+	4-	4-	4-	5
2	2+	3	3-	3-	3-	3-	3-	3-	5

**Table 2:** Clinical and biochemical features of the two patients at onset/after treatment

no	sex	age at onset (years)	disease duration (months)	myalgia	muscle weakness	other symptoms	laboratory data(U/L)				
							AST	ALT	LDH	CK	
1	F	25	48	+/-	+/-	abdominal pain, wheezing, fatigue/-	273/15	187/20	2364/186	2282/126	
2	M	14	4	+/-	+/-	weight loss, hypoglycemia, dysphagia, wheezing/-	1207/106	957/167	4102/558	627/41	

*AST* aspartate transaminase, *ALT* alanine transaminase, *LDH* lactate dehydrogenase, *CK* creatine kinase

**Fig. 1:**
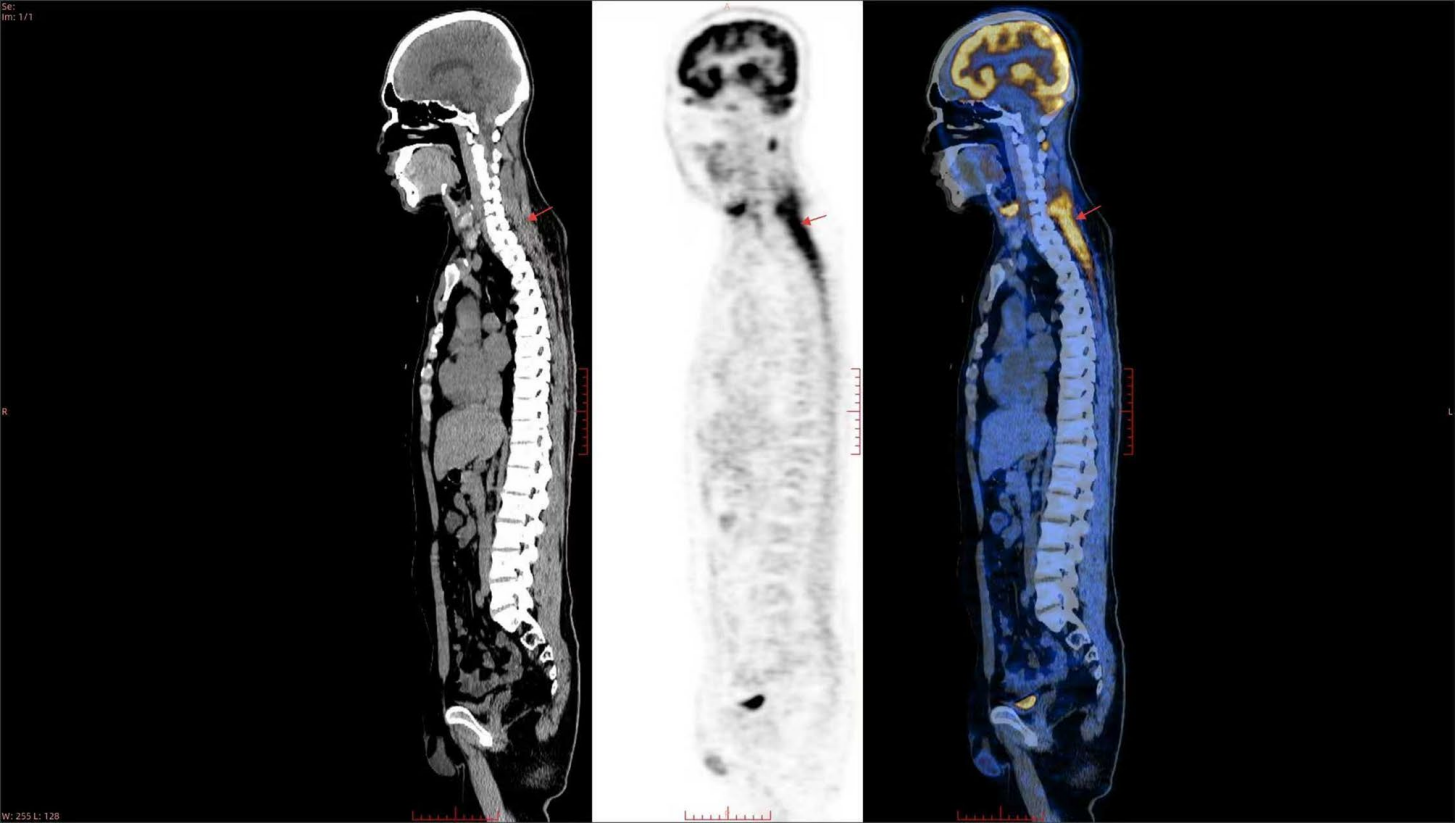
Case 1; ^18^F-FDG PET/CT. Diffuse metabolic activity was identified in the bilateral neck muscles (arrows)

**Fig. 2:**
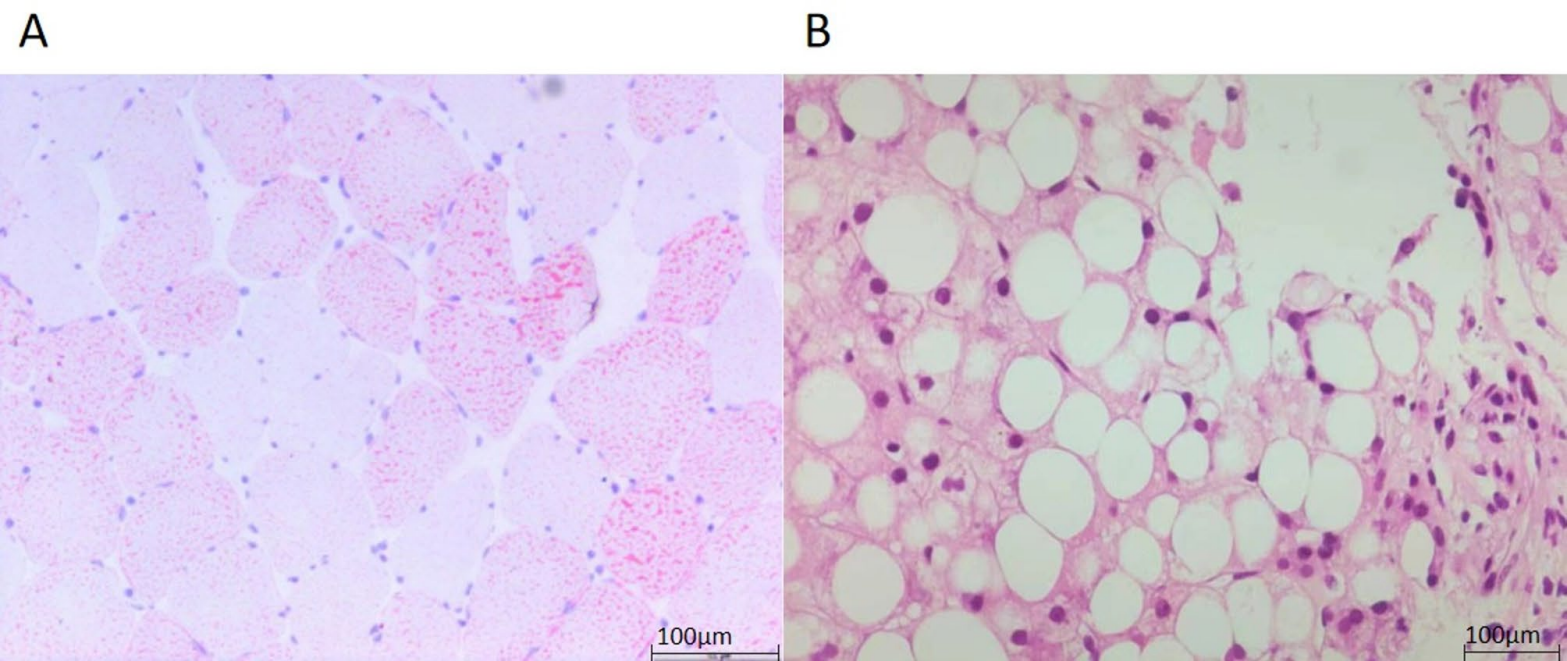
**A** Case 1; Diffuse lipid droplet accumulation in muscle fibers stained by Oil Red O. muscle tissue stained with the following all were negative results: hematoxylin and eosin, modified gomori triChrome, nicotinamide adenine dinucleotide dehydrogenase, succinate dehydrogenase, cytochrome C oxidase, neuron-specific enolase, periodic acid-schiff. **B** Case 2; Extensive microvascular steatosis was noted in the liver. The scale bars of 100 μm are displayed in the lower right corner

^18^F-FDG PET/CT demonstrated diffuse metabolic hyperactivity throughout the trunk and limbs muscles consistent with myositis (Fig. 1). The highest mean of the maximum standard uptake value (SUVmax) was 10.9 in the paraspinal muscles of the cervical spine (PC). Based on the PET/CT findings, the patient underwent muscle biopsy of the left quadriceps femoris, and Oil Red O staining verified lipid storage myopathy (Fig. 2A). Metabolic diseases were considered. His urine organic acid analysis was normal, but blood tandem mass spectrometry analysis detected decreased levels of a variety of acyl-carnitines (Table 3).

**Table 3:** Blood free carnitine. acylcarnitine. Amino acids and urine organic acids spectrum of the two patients*

no	increased urinary organic acid	abnormal acylcarnitine/amino acid	
		increased	decreased
1	-	C10	C2. C3. C16. C18. Glu. Ser
2	GA, MA,2-HGA	-	C0. Asp, Gln, Lys

*GA* glutaric acid, *MA* malic acid, *2-HGA* 2-Hydroxyglutaric acid, *Glu* glutamic acid, *Ser* serine, *Asp* aspartic acid, *Gln* glutamine, *Lys* lysine

*Blood amino acid and acylcarnitine mass spectrometry analysis was performed by using a liquid chromatography tandem mass spectrometer (3200MD, SCIEX, USA). Concentration levels of urine organic acids were determined by a gas chromatography mass spectrometry (GC/MS QP2020 Shimadzu, Japan)

**Fig. 3:**
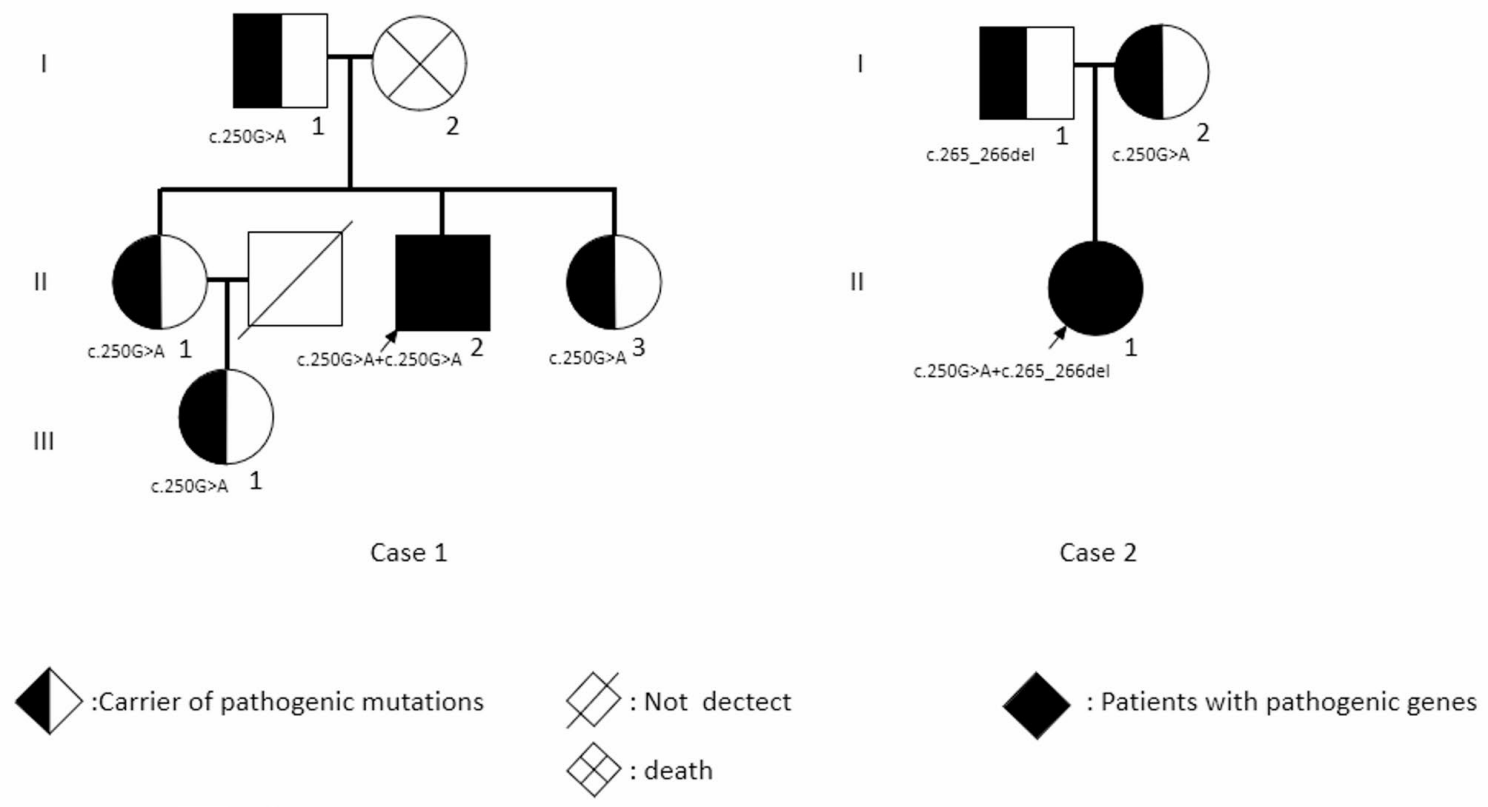
Genetic family trees of the two cases

Exome sequencing (ES) finally confirmed a diagnosis of MADD (Table 4). He had a homozygous missense mutation *ETFDH* NM_004453.4: c.250G > A (p. Ala84Thr). Sanger sequencing confirmed that his father, two sisters, and a 14-year-old niece all carried the heterozygous mutation at the same locus. However, none of the above relatives had developed MADD at the time of this report (Fig. 3). ES also showed he carried disomic Y chromosome, but the patient refused further verification of XYY syndrome.

**Table 4:** Molecular genetic characterization of the two patients

no.	gene	genetic relationship	exon	gene mutations	predicated effect	domain
1	*ETFDH*	paternal	3	NM_004453.4: c.250G > A	p. Ala84Thr	FAD
	*ETFDH*	NA	3	NM_004453.4: c.250G > A	p. Ala84Thr	FAD
2	*ETFDH*	paternal	3	NM_004453.4: c.265_266del	p. Gln89Valfs*6	FAD
	*ETFDH*	maternal	3	NM_004453.4: c.250G > A	p. Ala84Thr	FAD

*ETFDH* electron transfer of flavoprotein dehydrogenase, *NA* not Available, *FAD* flavin adenine dinucleotide

The patient was treated with riboflavin 30 mg daily and coenzyme Q10 30 mg daily and was advised to avoid strenuous exercise. Methylprednisolone and methotrexate were gradually tapered off over 1 month. After 6 months of treatment, no symptoms remained and all laboratory indices had returned to normal: AST 15 IU/L, ALT 20 IU/L, LDH 186 IU/L, and CK 126 IU/L (Table 2).

### Case 2

A 14-year-old female presented with a history of weight loss of 10 kg over the past 5 months. She also complained of recurrent hypoglycemia, proximal muscle pain, and progressive limb weakness in all four limbs for 2 months. She ultimately developed dysphagia and dyspnea after exercise. She was seen at a local hospital and her AST was 650 U/L and ALT was 482 U/L, and an upper abdominal computed tomography scan was reported consistent with severe fatty liver. There were no abnormalities in her family medical history.

On the admission physical examination, her liver margin was 2 fingers below the costal margin. The muscle strength of neck extensor, neck flexor, and proximal limbs were decreased (2+, 3, and 3, respectively) (Table 1). Biochemical tests indicated AST level of 1207 IU/L, ALT of 957 IU/L, LDH of 4102 U/L, and CK of 627 IU/L (Table 2). ANA, ENA series, and myositis antibody spectra were all negative.

^18^F-FDG PET/CT did not reveal any high metabolic tumor signs; however, there was increased metabolic activity in skeletal muscles consistent with myositis, and SUVmax was 6.8 in the paraspinal muscles of the cervical spine. Liver enlargement with significantly reduced density and metabolism diffuse indicated severe hepatic dysfunction. A liver biopsy was consistent with non-alcoholic fatty liver disease (NAFLD) (Fig. 2B). Subsequently, urine organic acid analysis displayed slightly elevated excretion of glutaric acid, malic acid and dihydroxy glutaric acid, while blood tandem mass spectrometry showed a decrease of several amino acids and some acyl-carnitines (Table 3). Finally, ES confirmed the diagnosis of MADD. She had a compound heterozygous mutation *ETFDH* NM_004453.4: c.250G > A (p. Ala84Thr), as in Case 1. However, the mutation in Case 2 was heterozygous, not homozygous. Sanger sequencing confirmed her mother also carried the heterozygous mutation at the same locus (Fig. 3). Another locus exhibited a frameshift mutation: *ETFDH* NM_004453.4: c.265_266del (p. Gln89Valfs *6). The frameshift mutation resulted in 2 bp deletions, leading to a frameshift in the transcribed sequence and premature termination of the coding sequence (Table 4). Mutation test indicated that the discovered variant sequence was highly conserved, and the mutation likely impaired protein function [13]. She also possesses a heterozygous frameshift variant: *SLC25A13* gene NM_014251.2: c.852_855del (p. Met285 Profs*2), which is associated with adult-onset citrullinemia type II.

The patient was treated with riboflavin 60 mg daily and levocarnitine 1 g daily. Methylprednisolone was gradually tapered over 1 month. After 6 months of treatment, no symptoms remained and all biochemical indicators had significantly improved: AST of 106 IU/L, ALT of 167 IU/L, LDH of 558 IU/L, and CK of 41 IU/L (Table 2).

## Methods

The detailed methods of pathological staining and mass spectrometry analysis are shown in Fig. 2; Table 3.

### Literature review of late-onset MADD with homozygous single locus missense mutations in ETFDH

A search of PubMed from 2000 to 2024 was performed through Medline Field using the terms: “MADD,” “glutaric aciduria type II”, “glutaric acidemia II”, “*ETFDH* mutation,” “riboflavin response”. The search results were further refined within titles and abstracts by the terms of “late onset” or “juvenile onset” or “adult onset”. A total of 77 studies were identified in the initial search, were thoroughly read, and filtered based on the following criteria: (1) description of the patient’s age and sex, with age > 28 days; (2) description of symptoms at presentation; (3) treatment with riboflavin and effectiveness of treatment; 4) availability of genomic mutation data with potential pathogenicity prediction; 5) absence of concurrent diseases at onset, including infection, tumor, or congenital disorders; 6) variants exclusively in *ETFDH*; 7) mutation type of homozygous single-locus missense mutation; 8) availability of full text. 

A total of 17 studies that met the inclusion criteria, and examination of the citations in these studies identified two additional articles that had not been captured in the initial search. These articles typically featured the term “*ETFDH*” in their titles. Finally, 19 studies of 32 patients with late-onset MADD were included in our review. The studies/cases were chosen because a single missense mutation in the homozygous state most clearly delineates the isolated impact of a genetic defect on the resultant ETF-QO protein dysfunction.

### Exome sequencing

The whole genome deoxyribonucleic acid (DNA) was extracted, captured, and a DNA library was constructed, which was subsequently subjected to high-throughput sequencing using the Novaseq 6000 platform (Illumina, USA). The data obtained had an average sequencing depth ≥ 90X across known exons and 5 bp upstream/downstream of the human genome, with at least 98% target sequences depth coverage ≥ 20X. Sequencing data were aligned to the human reference genome (GRCh37/hg19) from the University of California, Santa Cruz Genomics Institute Genome Browser database using Burrows-Wheeler Aligner software. This was followed by variant testing with the Genome Analysis Toolkit 3.7.

Subsequently, variant sites were annotated using Variable Effect Predictor software. Simultaneously, mutations were filtered based on genetic disease databases, variant databases, and large-scale population sequencing databases, such as Clinical Variant, Online Mendelian Inheritance in Man, HGMD, and Genome Aggregation Database using multiple recognized computer algorithms such as Sorting Intolerant From Tolerant, Protein Variation Effect Analyzer, and Polymorphism Phenotyping v2 to predict and classify the potential pathogenicity of mutations. High-throughput sequencing data was used for copy count variation analysis, and the variation rating parameters for the number of copies were based on the guidelines by American College of Medical Genetics and Genomics and Clinical Genome Resource.

### Sanger sequencing validation

Based on the high-throughput sequencing results, Sanger sequencing was used to verify the mutation loci of the family members. Primer-blast (https://www.ncbi.nlm.nih.gov/tools/primer-blast) online software was used to design the amplification of PCR. The products were sequenced on an ABI 3130xl sequencer (Thermo Fisher Co, USA).

## Discussion

### Adults tend to exhibit milder clinical symptoms compared to children

The first onset of MADD symptoms of the two cases presented herein was at age 29 and at age 14. As shown in Tables 1 and 2, the adolescent-onset patient (Case 2) had more severe clinical symptoms, lower muscle strength scores, and higher CK, AST, ALT, and LDH levels compared to the adult-onset patient (Case 1). In addition, ^18^F-FDGPET/CT revealed more pronounced metabolic activity indicative of myositis. As shown in Table 3, Case 1 exhibited no abnormalities in urinary organic acid excretion, whereas Case 2 displayed a slight elevation in organic acid excretion. Additionally, the types of altered amino acids and carnitine differed between the two cases. However, given that Case 2 and Case 1 have distinct types of genetic defects, it is not certain that juvenile patients have more severe conditions than adult patients.

Based on our literature search, we reviewed 32 late-onset cases with single-locus homozygous missense mutations in *ETFDH* (Table 5). The ratio of adult-onset to juvenile-onset cases was about 2:1. The survival rate and the proportion of RR-MADD in the adult-onset group were 100% (21/21) and 86% (18/21), respectively, while in the juvenile-onset group they were 73% (8/11) and 73% (8/11), respectively. All 3 juvenile-onset patients without RR-MADD did not survive (27%), whereas all 3 adult-onset patients without RR-MADD survived during the follow-up period. Thus, the prognosis for adult-onset patients appears to be better than for juvenile-onset patients.

**Table 5:** Case reports of homozygous mutation of the *ETFDH* gene of late-onset MADD

no.	age at onset	sex	syndrome	life outcome	cDNA mutation	amino acid change	REVEL score	domain	response to riboflavin	refs.
1	3y	F	delayed psychomotor, development, muscle weakness	alive	NM_004453.4: c.79 C > T	p. P27S	0.296	-	Y	Pollard Laura M. et al. [14]
2	6y	M	acidotic, nonketotic, hypoglycemic coma	alive	NM_004453.4: c.79 C > T	p. P27S	0.296	-	partial	Pollard Laura M. et al. [14]
3	14y	M	severe exercise intolerance, moderate muscle weakness	alive	NM_004453.4: c.245 C > T	p. S82F	0.929	FAD	Y	Béhin A. et al. [15]
4	19y	M	body weight loss, frequent abdominal pain, diarrhea	alive	NM_004453.4: c.250G > A	p. A84T	0.837	FAD	Y	Liang Wen-Chen et al. [16]
5	35y	M	muscle weakness	alive	NM_004453.4: c.250G > A	p. A84T	0.886	FAD	Y	Li Man et al. [17]
6	26y	M	episodic weakness, dyspnea	alive	NM_004453.4: c.250G > A	p. A84T	0.886	FAD	Y	Kuo Yih-Chih et al. [18]
7	23y	M	episodic weakness, exercise intolerance	alive	NM_004453.4: c.250G > A	p. A84T	0.886	FAD	Y	Kuo Yih-Chih et al. [18]
8	46y	M	progressive limbs muscle weakness, fatigue, exercise intolerance, intermittent abdominal distension, nausea, vomiting, dysphagia	alive	NM_004453.4: c.250G > A	p. A84T	0.886	FAD	Y	Chen Hai-Zhu et al. [19]
9	47y	-	muscle weakness, chewing difficulties, hypothyroidism	alive	NM_004453.4: c.523 C > T	p. R175C	0.812	FAD	Y	Lupica Antonino et al. [20]
10	12y	M	dysphagia, hypophonia, muscle pain, lost his head control, dyspnea, vomiting, seizures	alive	NM_004453.4: c.587 C > A	p. P196H	0.776	FAD	Y	Sophy Korula et al. [21]
11	24y	F	muscle weakness, exercise intolerance, dyspnea, thoracic pain	alive	NM_004453.4: c.769T > C	p. Y257H	0.949	FAD	Y	Béhin A. et al. [15]
12	50y	F	muscle weakness, exercise intolerance	alive	NM_004453.4: c.770 A > G	p. Y257C	0.848	FAD	Y	Lupica Antonino et al. [20]
13	27y	M	nausea and persistent vomiting,	alive	NM_004453.4: c.807 A > C	p. Q269H	0.886	FAD	Y	Arida Abdul et al. [22]
14	23y	M	recurrent abdominal pain, vomiting, impaired consciousness	alive	NM_004453.4: c.807 A > C	p. Q269H	0.953	FAD	Y	Lian Ling et al. [23]
15	34y	M	episodic myalgia, fixed weakness,	alive	NM_004453.4: c.998 A > T	p. Q333P	0.834	UQ	Y	Kuo Yih-Chih et al. [18]
16	3y	M	hepatomegaly, hypoglycemia, seizures at age 3 years, myopathy from age 8 years	dead(11y)	NM_004453.4: c.1074G > C	p. R358S	0.834	UQ	N	Olsen Rikke K.J et al. [18]
17	22y	M	acute hypoglycemia, metabolic acidosis, abdominal pain, liver function rapidly deteriorated	alive	NM_004453.4: c.1074G > C	p. R358S	0.886	UQ	Y	Soldath Patrick et al. [24]
18	6 m	M	muscle weakness	alive	NM_004453.4: c.1208 C > T	p. A403V	0.949	FAD	Y	Yamada Kenji et al. [7]
19	6 m	M	hypotonia,	alive	NM_004453.4: c.1208 C > T	p. A403V	0.89	FAD	Y	Mushimoto Yuichi et al. [25]
20	22y	F	hepatitis with vomiting, abdominal pain, fatigue, myalgia after delivery, exercise intolerance	alive	NM_004453.4: c.1366 C > T	p. P456S	0.929	-	Y	Béhin A. et al. [15]
21	35y	F	asthenia, muscle pain in lower limbs after delivery, exercise intolerance	alive	NM_004453.4: c.1366 C > T	p. P456S	0.856	-	Partial	Béhin A. et al. [15]
22	25y	M	agitation, vomiting, abdominal pain, rhabdomyolysis, hepatic steatosis, acute renal insufficiency, loss of weight, Muscle weakness	alive	NM_004453.4: c.1366 C > T	p. P456S	0.777	-	Y	Béhin A. et al. [15]
23	40y	M	rhabdomyolysis, liver dysfunction, hypotonia	alive	NM_004453.4: c.1367 C > T	p. P456L	0.89	-	N	Yamada Kenji et al. [7]
24	40y	M	muscle weakness, coma	-	NM_004453.4: c.1367 C > T	p. P456L	0.89	-	Y	Yamada Kenji et al. [7]
25	58y	M	mypotonia, muscle pain	alive	NM_004453.4: c.1367 C > T	p. P456L	0.89	-	N	Mushimoto Yuichi et al. [25]
26	5 m	F	moose stools, vomiting, lethargy, seizures, liver failure, myocarditis, multiple organ failure	dead(5 m)	NM_004453.4: c.1367 C > T	p. P456L	0.89	-	N	Keshri Swasti et al. [26]
27	35y	F	progressively worsening limb weakness, fatigue	alive	NM_004453.4: c.1514T > C	p. I505T	0.837	-	Y	Wang Chenyi et al. [27]
28	2 m	M	liver dysfunction, myopathy	dead(3y)	NM_004453.4: c.1519 T > G	p. Y507D	0.94	-	N	Yamada Kenji et al. [7]
29	42y	-	exercise intolerance, muscle weakness, chewing difficulties, dropped head	alive	NM_004453.4: c.1531G > A	p. D511N	0.93	-	Y	Lupica Antonino et al. [20]
30	24y	F	vomiting, respiratory insufficiency	alive	NM_004453.4: c.1544G > T	p. S515I	0.965	Fe-S	Y	Rosenbohm Angela et al. [28]
31	10 m	M	developmental delay	alive	NM_004453.4: c.1601 C > T	p. P534L	0.957	Fe-S	Partial	Yamada Kenji et al. [7]
32	12y	M	weakness, vomiting and hypertransaminasemia	alive	NM_004453.4: c.1658 A > G	p. Y553C	0.848	Fe-S	Y	Siano Maria Anna et al. [29]

*FAD* flavin adenine dinucleotide-binding domain, *UQ* ubiquinone Q-binding domain, *Fe-S* the iron-sulfur cluster domain

Our findings align with those of Yamada et al. who noted distinct differences between adult-onset and juvenile-onset patients, and proposed further subclassifying late-onset MADD (type III) into two subgroups based on in vitro probe acyl-carnitine technology: an intermediate (juvenile-onset) subgroup and a myopathic (adult-onset) subgroup [30]. Our case review confirmed that the prognosis for adult-onset patients is indeed better than that for juvenile-onset patients.

### MADD response to riboflavin and prognostic factors

Rare exome variant ensemble learner (REVEL, sites.google.com/site/revelgenomics/) combines the results of 13 prediction software in its assessment of missense mutation pathogenicity, and exhibits superior predictive performance than each single prediction tool and other composite scoring methods [31]. Though it is not a direct evaluation of the pathogenicity of a variant, it supplies a more refined classification method. As such, patients could be divided into 3 groups: pathogenic mutations group (REVEL score ≥ 0.85), deleterious mutations group (0.5 ≤ REVEL score < 0.85), and non-pathogenic mutations group (< 0.5). Considering that several previous reports have conducted typical pathogenicity tests on the mutations, we opted to use REVEL to evaluate the correlation between mutation pathogenicity and clinical severity across previously reported cases of late-onset MADD.

The REVEL analysis results of the previously reported 32 cases with late-onset MADD due to homozygous single-locus missense mutations are shown in Table 5.

Of the 32 patients, age range was from 2 months to 58 years and males were more common than females. Only three patients were reported to have died. The most common manifestation was myopathy (69%, 22/32), followed by gastrointestinal symptoms (34%, 11/32), liver involvement (25%, 8/32), neurological involvement (22, 7/32), and endocrine disorders (12% 4/32). There was no clear correlation between the affected organs and REVEL scores or functional domains, but no organ failure occurred in the non-pathogenic mutations group.

Twenty-one (66%) of the 32 patients had pathogenic mutations (REVEL score ≥ 0.85), 28% (9/32) had deleterious mutations (0.5 ≤ REVEL score < 0.85), and the remaining 6% (2/32) had non-pathogenic mutations (REVEL score < 0.5). None of the patients with non-pathogenic mutations died during follow-up. However, among those with pathogenic and deleterious mutations, mortality rates were similar: 10% and 11%, respectively.

Among the 30 patients with pathogenic or deleterious mutations (REVEL score ≥ 0.5), the patients with mutations in the FAD, UQ, Fe-S, and outside any structural domains exhibited riboflavin treatment effectiveness rates of 100%, 67%, 100%(1 partial response), and 40%(1 partial response), respectively, and their mortality rates of 0%, 33%, 0%, and 22%(2/9,1 is unknown alive or dead afterward), respectively. Patients with mutations outside any structural domains exhibited the lowest riboflavin efficacy rate, but the mortality rate was a bit lower than patients with mutations in the UQ. It seems a good prognosis is related to a good response to riboflavin treatment. As such, FAD and Fe-S demonstrated the best riboflavin response and prognosis compared to mutations in other regions.

According to the REVEL analysis, nonpathogenic mutations are associated with good prognosis. In the presence of deleterious mutations, both therapeutic response and prognosis are influenced by the affected structural domain. Deleterious mutations in FAD and Fe-S were associated with the best prognosis, while mutations in UQ and mutations outside domains had worse prognosis and worse riboflavin therapeutic response. Although these results are based on a small number of cases, our conclusion is consistent with the other studies to date, which indicates that mutations located within FAD and Fe-S typically result in good response to riboflavin and better prognosis. For example, Nanna et al. did an in vitro experiment using human HEK-293 cells, and confirmed four single locus missense mutations located in the FAD and UQ, some of which showed insensitivity to riboflavin treatment when located in the UQ [32]. This is consistent with our observation that patients with mutations in the UQ have a poorer prognosis. The likely propagation of mutation (p.Pro389Leu) in the UQ to the nearby FAD in *Rhodobacter sphaeroides* reported by Tania et al. further explains how certain loci may simultaneously affect the function of multiple domains [33]. Sara et al. reported that type III MADD had variations in 50% of FAD (73/145), 33% of UQ (47/145), and 12% of Fe- S (17/145); which means totally 62% variations occurred in FAD and Fe-S and this may be the reason why type III MADD is the mildest type [2]. However, all of the above studies did not analyze the single locus missense mutations in the UQ, as well as outside the functional domains. Further studies will be needed to explore how every single locus missense mutation in and outside the functional domains alter protein function.

### Significance of ^18^F-FDGPET/CT in diagnosing MADD

Magnetic resonance imaging (MRI) is the most used imaging method for diagnosing fatty deposition and muscle involvement. Short TI Inversion Recovery (STIR) sequences, where muscle tissue exhibits high signal intensity areas due to increased water content from cell lysis or fluid accumulation following inflammation are particularly useful [34]. The typical T1 relaxation time is approximately 300 milliseconds for fat, compared to around 1,000 milliseconds for most aqueous tissues [35]. This distinction enables clear visualization of fatty deposition in both subcutaneous and visceral compartments in cases of MADD [28].

However, Cases 1 and 2 were admitted to the rheumatology department due to suspected PM, and factors supporting a diagnosis of PM included persistently elevated muscle enzymes, muscle weakness, and myalgia, but no rash. PM increases the likelihood of developing cancer, hence ^18^F-FDG PET/CT is used to rule out malignancy in patients with suspected PM [36, 37]. Additionally, the method is useful for diagnosing myositis, evaluation of the extent of the disease, biopsy site identification, and the exclusion of interstitial lung diseases [38]. Therefore, we initially chose ^18^F-FDG PET/CT imaging for our 2 patients.

PM is clinically characterized by symmetrical proximal muscle weakness, but generally neck muscle involvement is absent or not severe [39, 40]. The study by Tateyama et al. assessed 16 muscle regions, including PC, and found only 1 out of 11 PM cases exhibited visually identifiable FDG uptakes (vFDG) in PC [41]. The study by Arai-Okuda et al. examined 18 muscle regions including PC, and found that 5 out of 10 cases had vFDG, but the SUVmax among the 5 cases was 2.90 [42]. However, the SUVmax in PC of Case 1 and Case 2 was 10.9 and 6.8, respectively, leading us to suspect they might not have PM. Subsequent pathological examination of tissue specimens confirmed that they had lipid storage myopathy. To date, there have been no reported cases or imaging studies of ^18^F-FDG PET/CT used in MADD, so it remains unknown whether the distribution of muscle involvement, vFDG, mean SUVmax, and mean SUV differ from PM. However, our cases suggest that ^18^F-FDG PET/CT can reflect muscle involvement and align with physical examination findings. Thus, it is possible that PET/CT may be a promising tool for assessing the extent of MADD myopathy.

### ES revealed coexisting hereditary abnormalities, which may overlap with the primary disease and present different clinical manifestations

ES identified a disomic Y chromosome in Case 1, but no confirmation studies were done. The patient had a tall stature (height 183 cm), was irritable, yet without intellectual abnormalities, motor coordination issues, social or emotional control disorders, or autism. Case 2 was found to have a genetic variant indicative of adult-onset type II citrullinemia. The disease can also manifest as fatty liver, hypoglycemia, and intrahepatic cholestasis, leading to jaundice, hypoproteinemia, coagulopathy, and hemolytic anemia. In adults, citrullinemia typically involves hyperammonemia and associated neuropsychiatric symptoms such as seizures, behavioral disturbance, memory impairment, disorientation, or consciousness disorders. Case 2 had a frameshift mutation consistent with pathogenicity for citrullinemia type II. However, she showed no signs of jaundice, had normal bilirubin and blood ammonia levels, and abdominal CT did not indicate cholestasis. Blood mass spectrometry analysis revealed no abnormal citrulline. While administration of sodium pyruvate is a supplementation option for citrullinemia patients, Case 2 was managed solely with riboflavin and L-carnitine, achieving normal liver function without the use of sodium pyruvate. Given the adult-onset nature of citrullinemia, further observation is necessary to ascertain whether the patient will develop the disease in the future.

We believe that ES is a practical tool for the rapid diagnosis of hereditary diseases. When the pathology report confirmed lipid storage myopathy, we did not know whether if it was MADD. A series of hereditary disorders can cause lipid storage myopathy. Moreover, we did not know whether this condition represented a single disease or multiple diseases, each of which might involve several mutations in several genes. Additionally, the blood and urine mass spectrum of our two patients showed only slight changes. Before proceeding with ES, we thoroughly consulted with the patients. They believed that investigating one gene at a time might delay the timing of treatment and agreed to undergo ES.

Although it is possible that the co-occurrences of other hereditary abnormalities in these two patients were incidental, it is necessary to investigate other potential hereditary conditions, considering the prognosis and heritability of hereditary diseases. Therefore, ES could be attempted in the future for rapid and comprehensive screening, though it may have regional limitations due to pricing and report timeliness.

## Conclusion

^18^F-FDG PET/CT is a promising imaging method for assessing the extent of MADD myopathy. In addition, ES can be employed for a rapid and comprehensive diagnosis of MADD and detection of any other coexisting genetic disorders. Mutation forms, the extent of mutation pathogenicity, and especially mutation locations in/out of functional structural domains are correlated with the effect of riboflavin treatment and prognosis.

## Supplementary Information

Supplementary Material 1.

## Data Availability

The datasets generated and/or analyzed during the current study are available in the ClinVar repository, [Accession: VCV000012028.44, VCV0000666174.19, VCV0000666174.19, VCV001069043.12, VCV000972080.11 and VCV000972080.11].
