# Supplementary material for: PET/CT and exome sequencing in late onset multiple acyl-CoA dehydrogenase deficiency: a case series and literature review
Source: BMC Med Genomics. 2025 Oct 21;18:166. doi: 10.1186/s12920-025-02210-8 (PMC12542620; doi:10.1186/s12920-025-02210-8)
Supplement: Supplementary file 1 — Supplementary Material 1. [file 12920_2025_2210_MOESM1_ESM.pdf]

# EDITORIAL CERTIFICATE

This is to certify that a medical editor who is an English native speaker associated with our company, Quicklyden Co., Ltd., has edited the manuscript entitled "PET/CT and exome sequencing in late onset multiple acyl-CoA dehydrogenase deficiency: a case series and literature review"

The medical editor who edited the manuscript has been being a professional editor and writer for several years and is a member of American Medical Writers Association.

Cordially,

Quicklyden Co., Ltd.

广州快立登翻译有限公司
